# Intracranial amelanotic melanoma: a case report with literature review

**DOI:** 10.1186/s12957-015-0600-z

**Published:** 2015-05-12

**Authors:** Jun Ma, Zhong Zhang, Shu Li, Xiaolin Chen, Shuo Wang

**Affiliations:** Department of Neurosurgery, Beijing Tiantan Hospital affiliated to Capital Medical University, China National Clinical Research Center for Neurological Diseases, Center of Brain Tumor, Beijing Institute for Brain Disorders and Beijing Key Laboratory of Brian Tumor, Beijing, 100050 China

**Keywords:** Central nervous system, Intracranial, Primary, Melanocytic lesion, Amelanotic melanoma

## Abstract

**Background:**

The incidence of primary central nervous system (CNS) melanocytic neoplasms is relatively low comparing to systemic ones. The performance of the tumor is variable. With poor clinical experience, the diagnosis and treatment of such tumors present to be a challenge. Amelanotic melanoma is an especially rare subtype. Only several cases have been reported.

**Case presentation:**

We report a case of intracranial amelanotic melanoma. Preoperative assessment revealed progressive right frontal mass. The patient underwent tumor resection. The pathologic analysis reported amelanotic melanoma of intermediate grade. The further examination of the whole brain and body was negative. The familial history was also negative. The patient recovered uneventfully and went on for radiotherapy and chemotherapy. After a follow-up period of 5 months, the patient was tumor-free.

**Conclusions:**

This is the second report about primary CNS amelanotic melanoma. We summarized characteristics of the primary CNS melanocytic lesions and amelanotic melanoma with review of the literature and review of cases of our department.

## Background

Primary melanocytic neoplasms of the central nervous system (CNS) belong to the tumors of the meninges. The incidence is less than 0.1%. Some may relate to certain neurocutaneous syndrome. The tumor can affect the entire CNS, with miscellaneous performances. The diagnosis is always difficult before the pathology result is available. And due to the potential of malignant transformation and dissemination, the identification of the original loci is even harder. So the clinical diagnosis and treatment must base on an overall evaluation of a patient. Primary amelanotic melanoma is a special subtype of the melanocytic neoplasm, which is especially rare. We want to share our experiences of primary CNS melanocytic neoplasms and amelanotic melanoma through our cases with review of the literature.

## Case presentation

### Medical history

A 64-year-old man presented to our department in December 2013 for progressive right frontal mass with dull aching for 9 months. His medical history was unremarkable except for 2 years of hypertension. The familial history was negative, with no neurocutaneous system disorders of the first grade relatives.

### Clinical examination

The vital signs were stable. A mass at the right frontal region could be palpated, about 5 cm in diameter, hard, fixed, with no obviously red, swollen, or tender of the superficial scalp.

### Imaging studies

**Figure 1:**
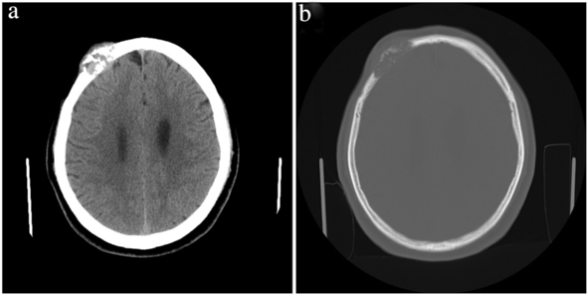
Preoperative CT scan of the patient. The lesion locates in the right frontal bone, isointense on CT scan, with prominent bone destruction. **(a)** CT scan of the soft tissue. **(b)** CT scan of the cranial bone.

Cranial CT scan showed an mixed iso-/hyperdensity mass in the right frontal cranial bone, about 60 to 198 Hu, measuring 34 × 21 mm, with clear margin. The peripheral bone appeared destructive absorption. The MR was contraindicated for metal implant (Figure 1).

### Surgery, pathologic result, and postoperative course

The patient underwent total tumor resection through right frontal approach on 24 December, 2013. The tumor eroded both the external and internal lamina of the cranial bone. The tumor was solid, soft, tender, pale white, rich blood supplied, and was adhesive to the underlying dura mater. The dura mater was yet intact, sparing the parenchyma of the cortex. The intraoperative rapid frost pathologic examination revealed malignant changes, and so after tumor resection, the adjacent dura mater was removed as well which was reconstructed with self aponeurosis. The peripheral bone was resected to the negative zone. The excision margin was about 2 cm.

**Figure 2:**
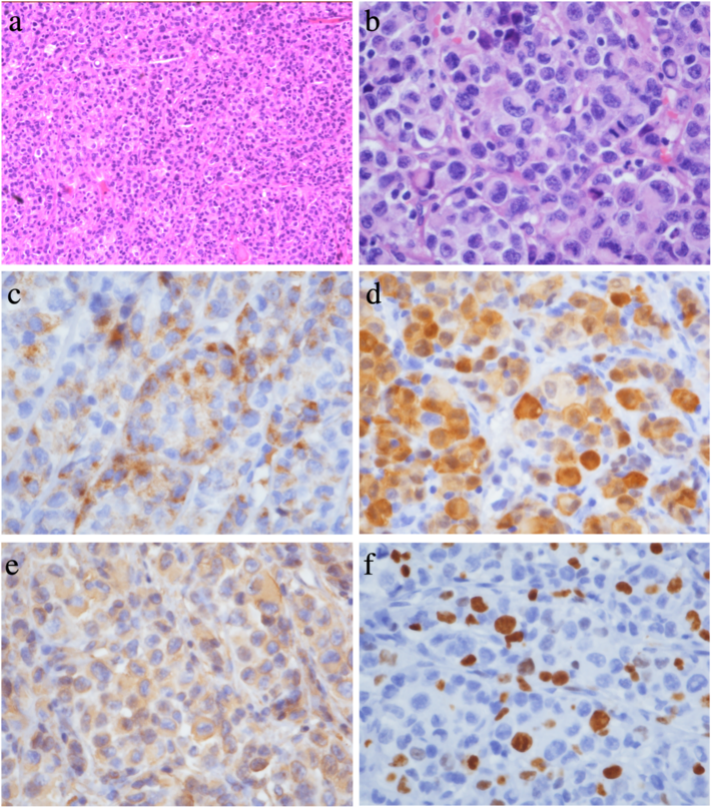
Pathologic results. **(a)** Pathology slice (hematoxylin-eosin stain, original magnification ×100) of the tumor. Nests of intermediate-grade tumor cells with intervening stroma can be seen. The tumor cells show clear to eosinophilic cytoplasm, with no obvious pigment. **(b)** Higher magnification (hematoxylin-eosin stain, original magnification ×400) of the tumor, showing prominent nuclear mitosis and atypia. **(c)** Higher magnification (original magnification ×400) of the tumor, showing HMB45 positive. **(d)** Higher magnification (original magnification ×400) of the tumor, showing S-100 positive. **(e)** Higher magnification (original magnification ×400) of the tumor, showing Vimentin-positive. **(f)** Higher magnification (original magnification ×400) of the tumor, showing Ki-67 index, was 20% to 30%.

The pathologic examination reported amelanotic melanoma of intermediate grade, with positive of HMB45, MelanA (sparsely), S-100, Vimentin, CD99, SMA (sparsely), and CD34, and negative of CK, EMA, CEA, CK5/6, CK8/18, TTF-1, SYN, CD56, CgA, Desmin, MyoD1, TFE3, PAS, and PR. The Ki-67 was 20% to 30% (±) (Figure 2).

The further examination includes CT scan of the chest, abdominal, and the pelvic region and the PET scan of the whole brain and body, which were all negative.

The patient was discharged 3 days after the operation with no complications and went on for adjuvant radiotherapy and chemotherapy (concomitant stereotactic radiosurgery + ipilimumab). For more than 5 months of follow-up, the patient recovers well with no recurrence or related sequelae.

### Review of cases and literature

We summarized the CNS melanocytic lesions of our neurosurgery department since 2008. Then we used the search engine of PubMed with keywords of melanoma or melanomatosis or melanocytoma or melanocytosis, and CNS and primary and further searched with keywords of CNS or brain or cranial or spinal, and amelanotic.

Thirty-two cases of CNS melanocytic lesions of our department were defined since 2008. Forty-three English reports of CNS melanocytic lesions and 14 reports about CNS amelanotic melanoma were searched. The details were summarized in the tables (Tables 1, 2, and 3).

**Table 1:** **Summary of CNS melanocytic lesions of our neurosurgery department since 2008**

**Case**	**Gender**	**Age (yr.)**	**Location**	**Pathology diagnosis**	**Preoperative differentiation** ^**a**^
1	M	46	Left temporal	Intermediate-grade melanocytic tumor	-
2	M	11	Right temporal	Malignant melanoma	-
3	M	45	Right frontal	Malignant melanoma	Metastasis? Lymphoma?
4	F	26	Right parasellar	Malignant melanoma	Schwannoma?
5	F	52	Left frontal	Metastatic melanoma	-
6	M	43	Right temporal	Meningeal melanomatosis	Metastasis? Pleomorphic xanthoastrocytoma?
7	M	47	Right CPA	Meningeal melanomatosis	Epidermoid cyst? Cavernous hemangioma?
8	M	24	Right lateral ventricle	Amelanotic melanoma?	-
9	F	19	Left parietal	Melanocytoma	Meningioma?
10	F	21	Right frontal, para-falx	Recurrent melanocytoma, with subarachnoid dissemination	Recurrent melanocytoma
11	M	38	Right petrosal	Meningeal melanomatosis	Schwannoma?
12	M	61	Multiple	Melanocytoma with meningeal melanomatosis	Melanoma? Metastasis?
13	M	20	Left temporal	Malignant melanoma	-
14	M	24	Right cerebellar	Malignant melanoma	Cyst?
15	M	47	Foreman magnum	Malignant melanoma, with bone invasion	Meningioma? Melanoma?
16	M	47	Left middle fossa	Malignant melanoma	Hemangiopericytoma? Meningioma? Melanoma?
17	M	45	Intradural extramedullary, C2-T1	Melanocytoma	Melanoma? Meningioma?
18	M	31	Right cavernous sinus	Melanocytoma	Melanoma? Cavernous hemangioma? Aneurysm?
19	M	46	Right middle fossa	Malignant melanoma, with bone and dura invasion	Meningioma?
20	M	20	Left pons	Malignant melanoma	Glioma? Meningioma?
21	F	41	Multiple, T6-L1	Melanocytoma	Neurofibrosis?
22	F	28	Right parietal occipital	Malignant melanoma, with subarachnoid dissemination	-
23	F	66	Left frontal	Malignant melanoma	-
24	M	27	Right interspinous foramen, C6	Melanocytoma	Melanoma?
25	M	39	Foramen magnum - C2	Melanocytoma	Lipoma?
26	M	36	Right frontal	Melanocytoma, with subarachnoid dissemination and parenchyma invasion	Melanoma? Lymphoma?
27	M	49	Right cerebellar tentorium	Meningeal melanomatosis	Meningioma?
28	M	17	Left parasallar - CPA	Melanocytoma	Epidermoid cyst?
29	M	26	Medulla	Intermediate grade melanocytic tumor	-
30	M	13	Right cerebellar tentorium	Malignant melanoma	Meningioma? Hemangiopericytoma?
31	M	47	Left temporal	Malignant melanoma	-
32	M	64	Right frontal bone	Amelanotic melanoma, with bone invasion	Eosinophilic granuloma? Giant cell osteoma? Metastasis?

M, male; F, female, yr., years old. ^a^With no clear preoperative diagnosis.

**Table 2:** **Review of the English literature of CNS melanocytic lesions**

**Author**	**Year**	**Nb**	**Primary/metastatic** ^**a**^	**Diagnosis**	**Other**
Foit *et al*.	2013	1	Primary	Melanocytoma	-
Lee *et al*.	2013	1	Primary	Meningeal melanomatosis	-
Schneider *et al*.	2013	1	Primary	Malignant melanoma	-
Reddy *et al*.	2012	1	-	Melanocytoma	-
Sutton *et al*.	2011	1	-	Leptomeningeal melanocytosis	The patient was suspected to represent a case of former fruste neurocutaneous melanosis.
Brunsvig *et al*.		1	-	Meningeal melanocytosis	-
Vij *et al*.	2010	1	Primary	Melanoma	-
Perrini *et al*.	2010	1	Primary	Malignant melanoma	-
Zadro *et al*.	2010	1	Primary	Diffuse meningeal melanomatosis	-
Nishihara *et al*.	2009	1	Primary	Malignant melanoma	-
Navas *et al*.	2009	1	-	Meningeal melanocytoma	Associated with a congenital nevus of Ota
Holfort *et al*.	2009	16	Metastatic	Melanoma	Out of review of primary uveal melanoma 2365 patients
Mathai *et al*.	2008	1	-	Intermediate grade meningeal melanocytoma	-
Tandon *et al*.	2008	1	Primary	Melanocytoma	-
Levidou *et al*.	2007	2	Unknown primary site	Leptomeningeal melanoma	-
Cajaiba *et al*.	2008	2	Primary	Neurocutaneous melanosis with CNS melanocytoma	Disseminated to the peritoneal surface by ventriculo-peritoneal (V-P) shunt system
Mekni *et al*.	2007	5	Primary	Malignant melanoma	-
Denaro *et al*.	2007	1	Primary	Melanoma	-
Bookland *et al*.	2007	1	Primary	Malignant melanoma	-
Kiecker *et al*.	2007	1	Primary	Neurocutaneous melanosis with meningeal melanoma	-
Oluigbo *et al*.	2006	1	Primary	Malignant melanoma	-
Chen	2003	1	-	Meningeal melanocytoma	-
Classen *et al*.	2002	1	-	Melanocytoma	-
Rivers *et al*.	2001	1	Primary	Dural melanoma	In association with ocular melanosis and multiple cutaneous blue nevi
Greco Crasto *et al*.	2001	1	Primary	Melanoma	-
Whinney *et al*.	2001	1	Primary	Malignant melanoma	-
Kobayashi *et al*.	2001	1	-	Malignant melanoma	-
Fathallah-Shaykh *et al*.	1996	1	Primary	Leptomeningeal melanoma	-
Eaves *et al*.	1995	1	Primary	Melanoma without diffuse leptomeningeal involvement	A variant of neurocutaneous melanosis
Rubino *et al*.	1993	1	Primary	Melanoma	-
Drake *et al*.	1993	8	Primary	Malignant melanoma of the leptomeninges	-
Singhal *et al*.	1991	1	Primary	Melanoma	-
Fish *et al*.	1990	1	Primary	Melanoma	-
Salisbury *et al*.	1989	1	Primary	Malignant melanoma	In association with a giant congenital melanocytic naevus of ‘bathing trunk’ distribution
Macfarlane *et al*.	1989	1	Primary	Malignant melanoma of the dura mater	-
Iglesias-Rozas *et al*.	1989	1	-	Disseminated melanomatosis of the CNS and other organs	-
Larson *et al*.	1987	5	Primary	Melanoma	-
Tamura *et al*.	1981	1	Primary	Leptomeningeal malignant melanoma	Originated from the basal aspect of the brain. The tumor showed not only spinal meningeal dissemination but also infiltration into the petrous bone and along the trigeminal nerve till it reached the submucosal tissue of epipharynx.
Pasquier *et al*.	1978	1	primary	Melanoma	With multiple metastases in the liver
Hayward	1976	27	20 Metastatic, 6 Primary	Malignant melanoma	-
Kaplan *et al*.	1975	1	Primary	Neurocutaneous melanosis with malignant leptomeningeal melanoma	-
Enriquez *et al*.	1973	1	Primary	Malignant melanoma	Pineal involvement in a patient with nevus of ota and multiple pigmented skin nevi.
Bergdahl *et al*.	1972	10	Primary	Malignant melanoma	-

CNS, central nervous system, the involved cases are all of the CNS, either intracranial or intraspinal; Nb, number of reported cases. ^a^Some cases have no clear evidence to be defined as primary or metastatic tumor.

**Table 3:** **Review of the literature of CNS amelanotic melanoma**

**Author**	**Year**	**Nb**	**Primary/metastatic** ^**a**^	**Diagnosis**	**Other** ^**b**^
Schulz *et al*. [6]	2012	1	Primary	Malignant amelanotic melanomas	(in German)
					Combined multiple intracranial and intraspinal primary malignant amelanotic melanomas
Cemil *et al*. [7]	2008	1	Metastatic	Amelanotic melanoma	-
Karakis *et al*. [8]	2007	1	Metastatic	Amelanotic melanoma	-
Jacob *et al*. [9]	2006	1	-	Amelanotic melanoma?	-
Li *et al*. [10]	2004	1	-	Amelanotic melanoma?	(in Chinese)
Ogawa *et al*. [11]	2003	1	Metastatic	Amelanotic melanoma	-
Shields *et al*. [12]	2002	1	Metastatic	Amelanotic melanoma	-
Isiklar *et al*. [13]	1995	16	Metastatic	Amelanotic melanoma	-
Schadendorf *et al*. [14]	1993	1	-	Amelanotic malignant melanoma	-
Takahashi *et al*. [15]	1990	1	Metastatic	Amelanotic melanoma	(in Japanase)
Krüger *et al*. [16]	1987	1	Metastatic	Amelanotic melanoma	Associated with a congenital nevus of Ota
Sunada *et al*. [17]	1986	1	Unknown origin	Amelanotic melanoma	(in Japanase)
					Out of review of primary uveal melanoma 2365 patients
Wagner *et al*. [18]	1981	1	-	Amelanotic melanoma	Amelanotic melanoma of the lung and brain
Schuknecht *et al*. [5]	1990	2	Primary	Meningeal amelanotic malignant melanoma	-

CNS, central nervous system, the involved cases are all of the CNS, either intracranial or intraspinal; Nb, number of reported cases. ^a^Some cases have no clear evidence to be defined as primary or metastatic tumor; ^b^literature in languages other than English is noted.

## Discussion

Primary melanocytic neoplasms of the CNS belong to the tumors of the meninges, further classified as diffuse melanocytosis, melanocytoma, malignant melanoma, and meningeal melanomatosis. The incidence is 0.06% to 0.1% of melanocytoma and 0.005/100,000 of melanoma. Other subtypes are rare. There is a slight female predisposition, with the ratio of F:M = 1.5:1. The age range of melanocytoma is 9 to 73 years, most frequently from 45 to 50 years, and of the primary nodular melanoma is 15 to 71 years, averaging 43 years. The symptoms and signs are secondary to either the local effects on the CNS parenchyma or the accompanying hydrocephalus. The rapid progress with increasing ICP resulting in irritability, vomiting, lethargy, seizures, and so on may suggest malignant transformation. The diagnosis of melanocytic lesions relies on the histopathological examination. Most benign and malignant melanocytic lesions display melanin pigment distributed within tumor cells, tumor stroma, and the cytoplasm of tumoral macrophages (melanophages). Tumor with CNS invasion or elevated mitotic activity is classified as intermediate grade melanocytic neoplasms. Malignant melanoma is more pleomorphic, has more anaplastic nuclei, higher cell density, and unequivocal invasion or coagulative necrosis. Rare melanocytomas and occasional primary melanomas will not demonstrate melanin pigment, complying with ‘amelanotic’ Amelanotic melanoma may arise from normal melanoma with amelanotic transformation or detergent of the tumor cell. Histopathological and immunohistochemistry examinations are highly sensitive to amelanotic melanoma. The neuroimaging performance depends on the content of melanin. Isiklar *et al*. [1] classified the MR performance into four groups: a) The melanotic group, with hyperintense on T1 and hypointense on T2. b) The amelanotic group, with iso-/hypointense on T1 and iso-/hyperintense on T2. c) The mixed group, suiting neither the two criteria. d) The hemorrhagic group, with characteristics of intra-/peri-tumoral hemorrhage. Gaviani *et al*. [2] reported that the T1 signal has positive correlation with the content of melanin, while T2 signal has no such correlation but is more sensitive to tiny loci.

Early in 1980s, Willis *et al*. pointed out that the diagnosis of ‘primary’ has to meet three critical conditions: a) The skin or eyeballs are negative of melanoma. b) The skin or eyeballs have no history of melanoma resection. c) The internal organs are negative of metastasis of melanoma. Savitz [3] thought that the systemic melanocytic lesions are very likely to metastases to CNS while the primary loci are quite indolent. Winkelman *et al*. [4] reported that 44% of all melanocytic lesions have CNS metastasis at autopsy, so that the ‘primary’ can only be confirmed after autopsy. So every melanocytic neoplasm may be a systemic disease, and the ‘primary CNS melanocytic lesion’ is just a subtype with CNS as the single manifestation clinically. There are approximately more than 60 papers describing primary CNS melanocytic lesions, with about 40 are in English, dated from 1972 to 2013. The number of cases ranged from one to ten. The symptoms are various according to the location of the tumor and so are the neuroimaging performance relating to the content of the melanin, as mentioned before. About 14 papers referred to CNS amelanotic melanoma, from 1981 to 2012. Most are case reports, including two primary intraspinal and two primary meningeal amelanotic melanoma, 22 intracerebral metastasis, one intraventricular metastasis, and three cases with no details. Schuknecht *et al*. [5] reported two primary meningeal amelanotic melanoma in 1990, serving as the only paper describing primary CNS amelanotic melanoma. There are totally 32 primary or metastatic CNS melanocytic lesions of our department since 2008. The cases diagnosed earlier are not included because of the controversial diagnosis criterion before the 2007 WHO classification which are widely adopted. The location involves all the CNS, from supratentorial to intraspinal region. Due to the unspecific resemblance, there were only few cases which were diagnosed before surgery, and the differentiations were quite variable. The most common differentiations are tumors of the meninges, which belong to the same group of the melanocytic lesions, such as meningioma and the mesenchymal tumors. The hematopoietic neoplasms, cavernous hemangioma, and even the cystic lesions are also involved especially when there are hemorrhage, cystic changes, or necrosis, and so on. One of them was suspected as amelanotic melanoma. Our case is the second one with definite diagnosis of amelanotic melanoma. The clinical characteristics of amelanotic melanoma are similar to other melanocytic lesions, with the neuroimaging performance as the major difference. Also, during the operation, the amelanotic melanoma appears more pale white, and the microscopic examination reviews the lacking of melanin. As the overall examinations were all negative, the primary intracranial amelanotic melanoma was confirmed.

The prognosis of amelanotic melanoma is not quite different from other CNS melanoma. Melanocytoma lacks anaplastic features, but a few undergo local recurrences. Intermediate grade melanocytic tumors typically invade the CNS. A rare example of malignant transformation of a melanocytoma has been reported. Malignant melanoma is highly aggressive and radioresistant, has poor prognosis, and prone to metastasize. The prognosis of diffuse melanosis is poor even in the absence of histologic malignancy. The prognosis of the primary CNS melanocytic lesions appears to be better than metastatic examples. For our case of primary intracranial amelanotic melanoma, total resection was achieved as well as excessive removal of invaded cranial bone and adjacent meninges. The experience of postoperative adjuvant therapy is quite poor. We adopted the radiotherapy and chemotherapy regimen of concomitant stereotactic radiosurgery + ipilimumab, referring to the NCCN guidelines for melanoma (2.2015 version). With adjuvant radiotherapy and chemotherapy, the patient is tumor-free 5 months later and is still under follow-up.

## Conclusions

CNS amelanotic melanoma is quite rare. The clinical characteristics are similar to other melanocytic lesions, with the neuroimaging performance as the major difference. The prognosis of amelanotic melanoma is not quite different from other CNS melanoma and appears to be better than the metastatic cases, particularly if localized and complete resection is possible.

## Consent

Written informed consent was obtained from the patient for the publication of this case presentation and accompanying images. A copy of the written consent is available for the review by the Editor of this journal.
